# Mixed Contaminants: Occurrence, Interactions, Toxicity, Detection, and Remediation

**DOI:** 10.3390/molecules27082577

**Published:** 2022-04-16

**Authors:** Anirban Goutam Mukherjee, Uddesh Ramesh Wanjari, Mohamed Ahmed Eladl, Mohamed El-Sherbiny, Dalia Mahmoud Abdelmonem Elsherbini, Aarthi Sukumar, Sandra Kannampuzha, Madurika Ravichandran, Kaviyarasi Renu, Balachandar Vellingiri, Sabariswaran Kandasamy, Abilash Valsala Gopalakrishnan

**Affiliations:** 1 Department of Biomedical Sciences, School of Bio-Sciences and Technology, Vellore Institute of Technology, Vellore 632014, Tamil Nadu, India; mukherjee1anirban@gmail.com (A.G.M.); uddeshwanjari786@gmail.com (U.R.W.); sandrajacobkannampuzha@gmail.com (S.K.); madurika2902@gmail.com (M.R.); 2 Department of Basic Medical Sciences, College of Medicine, University of Sharjah, Sharjah 27272, United Arab Emirates; meladl@sharjah.ac.ae; 3 Department of Basic Medical Sciences, College of Medicine, AlMaarefa University, Riyadh 71666, Saudi Arabia; msharbini@mcst.edu.sa; 4 Department of Clinical Laboratory Sciences, College of Applied Medical Sciences, Jouf University, Sakaka P.O. Box 2014, Saudi Arabia; dmelsherbini@ju.edu.sa; 5 Department of Anatomy, Faculty of Medicine, Mansoura University, Mansoura 35516, Egypt; 6 Department of Integrative Biology, School of Bio-Sciences and Technology, Vellore Institute of Technology, Vellore 632014, Tamil Nadu, India; aarthisukumar27@gmail.com; 7 Centre of Molecular Medicine and Diagnostics (COMManD), Department of Biochemistry, Saveetha Dental College & Hospitals, Saveetha Institute of Medical and Technical Sciences, Saveetha University, Chennai 600077, Tamil Nadu, India; kaviyarasirenu.92@gmail.com; 8 Human Molecular Cytogenetics and Stem Cell Laboratory, Department of Human Genetics and Molecular Biology, Bharathiar University, Coimbatore 641046, Tamil Nadu, India; geneticbala@buc.edu.in; 9 Department of Biomass and Energy Conversion, Institute of Energy and Environmental Engineering, Saveetha School of Engineering, Saveetha Institute of Medical and Technical Sciences, Saveetha University, Chennai 602105, Tamil Nadu, India; sabariswaran14@gmail.com

**Keywords:** contaminant, biomarkers, pollution, remediation, microorganisms

## Abstract

The ever-increasing rate of pollution has attracted considerable interest in research. Several anthropogenic activities have diminished soil, air, and water quality and have led to complex chemical pollutants. This review aims to provide a clear idea about the latest and most prevalent pollutants such as heavy metals, PAHs, pesticides, hydrocarbons, and pharmaceuticals—their occurrence in various complex mixtures and how several environmental factors influence their interaction. The mechanism adopted by these contaminants to form the complex mixtures leading to the rise of a new class of contaminants, and thus resulting in severe threats to human health and the environment, has also been exhibited. Additionally, this review provides an in-depth idea of various in vivo, in vitro, and trending biomarkers used for risk assessment and identifies the occurrence of mixed contaminants even at very minute concentrations. Much importance has been given to remediation technologies to understand our current position in handling these contaminants and how the technologies can be improved. This paper aims to create awareness among readers about the most ubiquitous contaminants and how simple ways can be adopted to tackle the same.

## 1. Introduction

In the past few years, environmental pollution by mixed contaminants released due to higher living standards has increased concern for its control. Although it is essential to eliminate toxins from a place, it is frequently not possible owing to technological, environmental, geographical, social, or budget constraints.

Most emerging pollutants arise from anthropogenic activities and mostly end up in the environment in some way. When the residues are released into the environment, all the possible emerging pollutants are subjected to various degradation processes to eliminate them [1]. Certain synthetic chemicals can be degraded and reduce the population of xenobiotics and the formation of emerging pollutants. Other chemicals and emerging pollutants under the stress of biological processes will try to change into a transformation product based on the environment (soil, groundwater, drinking water, surface water, wastewater, treatment plants, or sediments) in which they are present [2,3]. These transformed products exhibit more significant toxicity and enhanced persistence when compared to their parent compound. They may also gain new properties when they transform. This chemical transformation follows either the biotic or abiotic transformation process. The biotic process results in transformation by increasing the polarity of the compounds [4].

In contrast, the abiotic process includes photolytic reactions resulting in many transformation products, which can be even more toxic. Certain transformed products retain their property or lose their toxicity and anti-microbial activity. These chemicals bypass wastewater treatment but cannot withstand the biodegradation process that could eliminate them from the environment [5,6].

### 1.1. Mode of Action of the Interacting Pollutants

The mode of action begins with the interaction of the toxicant with the receptor, resulting in functional and anatomical changes in the organism that cause lethal or sub-lethal complications [7]. For a toxicant to have a considerable effect, a few factors such as bioavailability, toxicokinetics, biological transformation, and toxicodynamics have to be considered.

#### 1.1.1. Bioavailability

Bioavailability is defined as the level of availability of a metal or chemical toxicant, either individually or in mixtures, to cause a synergistic or antagonistic change in an exposed individual. It greatly depends on the toxicants’ partitioning behavior or binding strength to the receptors [8]. The bioavailability of a toxicant is evaluated based on its physical and chemical interactions in the spatial distribution of the environment. The presence of surfactants increases bioavailability through dispersion and pollutants in the environment. Nanomaterials have been reported to act as carriers of metal toxicants, making them more available to organisms [9,10]. Non-carbon nanomaterials such as layered double hydroxides, iron oxide magnetite nanoparticles, nano-polymer composites, metal oxide nanomaterials, and nanomembranes/fibers have all been implicated in heavy metal-contaminated water and in environmental cleanups [11].

#### 1.1.2. Toxicokinetics

Toxicokinetics refers to whole-body kinetics such as absorption, distribution, metabolism, and excretion when a toxicant is exposed to an organism. The toxicant enters an organism and is then diffused to various body parts via several transporters [12,13]. The ingested toxicant is metabolized into minor or less harmful metabolites, which can then be excreted or bioaccumulated in various parts of the body. Biotransformation, excretion, and storage at various places throughout the body remove toxicants from the systemic circulation. Xenobiotics are removed from the bloodstream and returned to the external environment by urine, feces, or breath [12,13].

#### 1.1.3. Biological Transformation

The uptake of mixed contaminants occurs via their interaction with the active site of the organism during the exposure. This uptake is governed by the biological membrane, in which damaged membranes do not allow the metals to pass through intracellular compartments; relative transporters can also eliminate them [14]. In the case of heavy metals, surface transporters act as the potential active sites, resulting in the uptake of the chemicals, which is called biological transformation. During biological transformation, the mixed toxicants are broken down into more polar components by adding polar groups such as amino, hydroxyl, and carboxyl, thus making them easy to excrete [15,16]. Biological transformation is carried out in three phases. Phase-1 involves the cytochrome P450 enzyme-mediated oxidative, reductive, and hydrolytic transformations of a chemical or metal toxicant. Phase-2 involves the transferase enzyme-mediated conjugative transformation of compounds that enhances the hydrophilicity of toxicants. Phase-3 involves the transporter protein-mediated absorption, distribution, and excretion of metabolites [17,18]. In rare cases, the breakdown of the parental compound to minor metabolites can produce a toxic compound and cause lethal changes. However, biological transformation is highly involved in detoxification [19] or the reduction of the toxicity of the toxicant, thereby maintaining biochemical homeostasis [14].

#### 1.1.4. Toxicodynamics

Toxicodynamics refers to the active interactions between a toxicant and a target, resulting in various structural and functional dysfunctions. It has been reported that mixed contaminants can have multiple or unknown modes of action that interact with the target receptors [20].

The main objective is to give a clear idea about the mixed contaminants and their complex products, about the mechanism adopted by these contaminants to form the complex mixtures that lead to the rise of a new class of emerging contaminants and result in severe threats to human health and the environment, and about innovative methods to detect and eliminate these contaminants from the environment.

## 2. Fate as well as Behavioral and Pathophysiological Aspects of Mixed Contaminants

### 2.1. In Soil

Soil has been constantly exposed to a variety of contaminants and their cocktails in various concentrations (for example, micro/nano plastics, heavy metal accumulation [21], organic/inorganic chemical exposure, which mainly includes pesticides and their monomer mixtures), which contaminate the soil to a greater extent and can also be transported within the soil and transfer the mixed contaminants by integrating into the food chain and passing on through different trophic levels, causing health effects on the organisms exposed to them [22].

#### 2.1.1. Pathophysiology Involved in Micro/Nano Plastic Exposure

**Figure 1:**
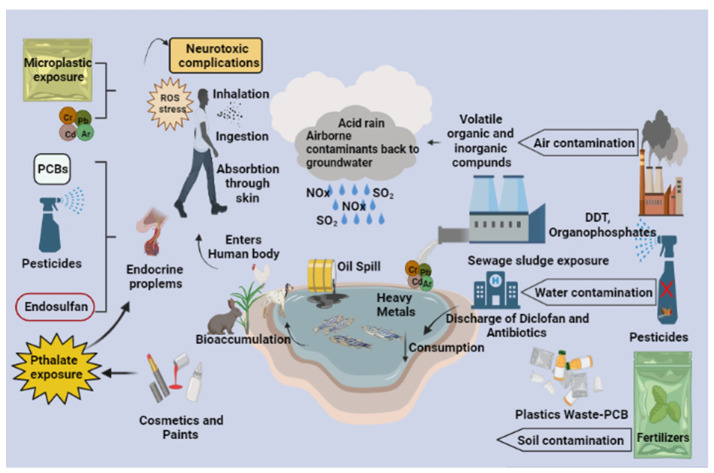
Influence of anthropogenic activities leads to the accumulation of pollutants in the environment. Waste disposed by factories and pharmacies containing mixed contaminants enters the food chain and bioaccumulates in higher organisms. These pollutants in living organisms alter several biological mechanisms and threaten normal development.

Micro/nano plastics mainly arise from the manufacturing industries that produce products using micro/nano plastic material. The unsaturated compounds interact with UV radiation, which can further trigger a cascade of chemical reactions, eventually leading to the breakdown of long-chain polymers and the formation of micro and nano plastics. Basic plastic wastes include low and high-density polyethylene, polyamide, polyimide [9,10], polypropylene [23,24], polyvinyl chloride (PVC), polystyrene (PS), polyamide, polyethylene tetra phthalate, and [25,26] that can change the arrangement of the polymers due to environmental stress gaining an added advantage of intercalating themselves and other contaminants, thus emerging as a potential threat with enhanced toxicity as a result of their modification [12,15]. When the micro/nano plastic debris is ingested, it can cause gut inflammation, intestinal block, tissue abrasion, neurotoxic and oxidative stress, and sometimes genotoxic and even carcinogenic effects. It can reach the gut epithelium, triggering the immune system, activating the cytotoxic T-cells and B-cells, which cross the threshold and cause inflammatory gene expression that induce inflammatory cytokines such as IL-1, TNF-ɑ, and TGF-β; this can lead to tissue abrasion and pulmonary fibrosis in case of nano plastic inhalation [25,26]. With less than 50 nm in diameter, nano plastic debris can penetrate the skin and reach blood circulation [25,26]. Nano plastics can even cross the blood–brain barrier and cause neurotoxic effects. These cascades of inflammatory signals can also trigger the tumor cells that can end up causing lung carcinoma when inhaled and gastric cancer when ingested. Direct ingestion of micro and nano plastics can entirely or partially block the gastrointestinal tract, leading to rapid death [27,28]. If oriented incorrectly, it can pass through the gut, perforate the stomach, and accumulate in the intestines, interrupting the gut functions, as it has been observed in marine turtles. It can sometimes cause ulcerations and disrupt the receptor signals that send the feeling of satiation to the brain, thus resulting in decreased appetite and a reduced drive to search for food in animals (Figure 1) [27,28].

#### 2.1.2. Pathophysiology Involved in Organic and Inorganic Chemical Exposure

Organic contaminants include chemicals mainly originating from industrial outlets such as oil and petroleum spills, concrete pollution, organic pesticides, synthetic herbicides, and fungicides, either in a single form or in a mixture [29]. Organic contaminants can alter the body’s metabolism by inhibiting or activating enzymes that can alter gene regulation, inducing changes in vital systems such as the endocrine and nervous systems. Most organic molecules produce DNA damage via ROS production and are probable carcinogens [30]. Certain chlorinated organic pollutants such as dioxin can metabolize estrogen by inducing the cytochrome P450 enzyme, exhibiting anti-estrogenic activity by effectively binding to the aryl hydrocarbon receptor [31]. Some organic contaminants have also been found to have anti-androgenic activity by reducing the production of male sex hormones [31]. Persistent organic contaminants such as PCBs, dioxins, DDE, and hexachlorobenzene have been involved in the onset of type-2 diabetes due to alterations in gene regulation. Chemical contaminants have also been reported to cause cardiovascular disease in vulnerable people, mainly because of the synthesis of ROS and lipids, which alters the immune response and causes atherosclerosis by inducing damage to the endothelial cells [32,33].

Inorganic chemical contaminants generally include metals and simple chemicals widely found in low-lying areas of urban lands [29]. Occupational exposure to these contaminants occurs mainly through inhalation but can also be ingested or enter through the skin. Inhalation of radioactive gases such as radon and other trace elements is highly reactive [34,35]. On inhalation, they release alpha and beta particles that have been reported to cause lung cancer in uranium miners and nearby residents [36]. The exact mechanism applies to asbestos, which is reported to be one of the primary reasons for the onset of lung cancer and other acute health effects such as shortness of breath, tightness in the chest, asthma, and dry cough. Asbestos can remain in the lung for many years and cause parenchymal asbestosis, asbestos-related pleural abnormalities, lung carcinoma, and pleural mesothelioma [34,35]. Cadmium is primarily present in irrigation water for plants. It enters the food chain and causes liver or kidney damage and low bone density. Other metallic pollutants such as lead, mercury, and others are also equally toxic, causing significant damage to the CNS; these have been reported to be carcinogenic when ingested directly or during prolonged exposures [37].

Heavy Metal Mixture Exposure

**Figure 2:**
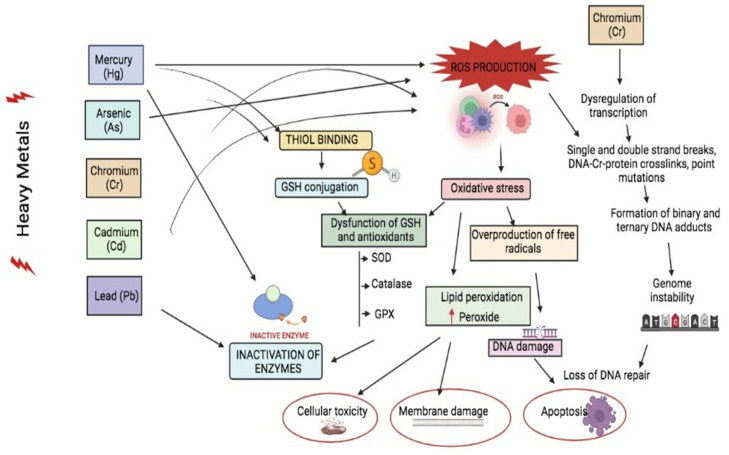
Toxic mechanisms and effect of the cellular level of heavy metals Hg, As, Cr, Cd, and Pb. These heavy metals provoke ROS production inside the cell and cause an imbalance of ROS and antioxidant production, thus causing cellular damage via inactivation of enzymes, apoptosis, genome instability, and DNA damage. Up arrow (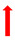) indicates increases.

Heavy metals and their mixture enter the environment from various sources, including natural and anthropogenic activities. Heavy metals such as Cr [38,39,40], Cd, Pb, Ni, Hg, and metalloids such as As and others, present alone or in mixtures, can invade the human body by adsorption, diffusion, ingestion, inhalation, or dermal contact, mostly through occupational exposures that have enormous toxic effects such as cytotoxicity, neurotoxicity, hepatotoxicity, nephrotoxicity, and even carcinogenicity (Figure 2). The liver stands to be the prime organ to metabolize every reaction in the body [41]. It was estimated that long-term exposure to a metal mixture containing Cd, Pb, and As interrupts phase-I and II reactions, resulting in the production of unsaturated reactive species that cause hepatotoxicity and various side effects in the liver cells. External exposure to Hg, Pb, As, and Cd has been revealed to cause neurotoxicity, apoptosis (programmed cell death), excitotoxicity (affecting neurotransmitter storage and release and modifying neurotransmitter receptors); mitochondria, second messengers, cerebrovascular endothelial cells, and both astroglia and oligodendroglia are all direct neurotoxic effects of these heavy metal exposure [42,43,44]. The level of arsenic in US drinking water is less than 5 g/L. It is estimated that 350,000 people will consume water with a concentration of more than 50 g/L [45]. Cd has produced considerable oxidation of the peptidyl cysteines of proteins regulating the actin cytoskeleton and increased filamentous actin polymerization at concentrations similar to those found in the environment (0.5–1 M) [46]. Pb is a very hazardous substance that has negative effects on human neurological, biochemical, and cognitive capabilities. Pb poisoning has an international level of concern of 10 g/dL in the blood [47]. Another study found that methylmercury chloride caused CNS damage in rats at varying levels of 0.05, 0.5, and 5 mg/kg Hg as compared to a control group of rats given normal saline. Increased expression of the c-fos protein in the cortex and hippocampus as a key signal transduction channel revealed CNS injury. In the treated rats, Hg build-up in the brain was also detected [48].

#### 2.1.3. Pathophysiology Involved in Pesticide Exposure

**Figure 3:**
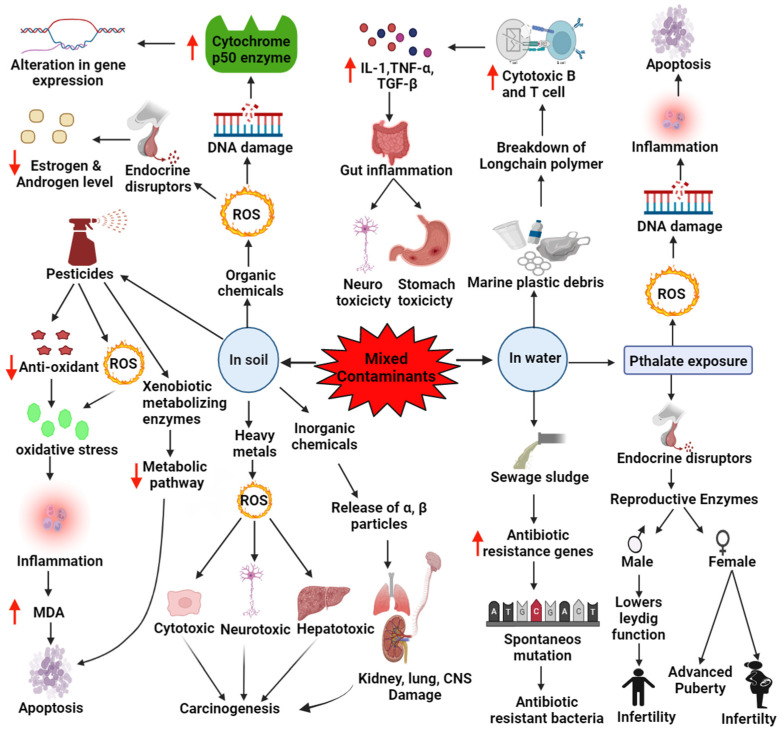
Effects of mixed contaminants in soil and water. Pathophysiology is followed by different contaminants in humans, eventually leading to cancer. Water contaminated with phthalate and its exposure to humans has resulted in various reproduction-related complications in males and females. Up arrow (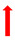) indicates increases and down arrow indicates (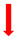) decreases.

Many combinations of pesticides have been reported to induce changes in the metabolic pathway, alter lipid metabolism, cause oxidative stress, induce a disturbance in the homeostasis, disrupt endocrine glands, cause malformations, affect fetal growth, and can even sometimes be carcinogenic mutagenic when alterations in DNA occur [49]. A total of 90% of pesticides used on cereal and corn have been reported to alter the metabolism of glucose, lipids, amino acid profile, and acetylcholinesterase. In toxicology laboratories, a substance’s toxicity is determined and stated in numerical terms, such as LD50 or LC50 (lethal dose or concentration of 50%, i.e., the dose or concentration at which a material will kill 50% of a reference organism). Toxicity is indicated by an LD50 of less than 500 mg/kg. Toxicity is moderate when the LD50 is 500–1000 mg/kg. Low toxicity is indicated by an LD50 of 1000–2000 mg/kg [50]. A combination of phenylacetlyglycine and hippurate has been observed to disrupt gut microbiota, which can also be excreted in the affected person’s urine. Metabolic changes in the pathways such as amino acid metabolism in urea cycle disorders can occur when the patients are exposed to pesticide cocktails containing atrazine, chlorpyrifos, and endosulfan [51]. A study demonstrated that the single and combined application of herbicides and insecticides can pose a severe threat to several taxonomic groups, but no change was observed in the metamorphosis. Insecticides cause 99% mortality in larval amphibians, giving rise to free radicals’ ROS production, causing inflammation responsible for disturbance in the metabolic pathway. Several data on combined pesticide exposure containing organophosphates [52,53], organochlorines [54,55,56,57], pyrethroids, and carbamates, alone or in combination, show how this can induce liver and brain abnormalities in different model organisms, causing metabolic and neurological disorders. These pesticides alone or in combination cause mitochondrial damage due to the depletion of antioxidants, the production of ROS, and the upregulation of xenobiotic-metabolizing enzymes, triggering lipid peroxidation in the cells of the exposed individual. This releases inflammatory cytokines that impact on insulin receptor phosphorylation, leading to the impairment of the insulin signal transduction (Figure 3) [58,59].

### 2.2. In Water

#### 2.2.1. Pathophysiology Involved in Sewage Sludge

Sewage sludge refers to the biosolids primarily present in the water from wastewater treatment plants [6,60]. It consists of semi-solid particles that may contain various compounds such as nutrients, organic matter, pathogens, micro/nano plastic materials, and organic/inorganic contaminants that are discharged into the sewer system without proper treatment from the domestic areas and industries, and which end up in the nearby water bodies and the streets. It includes heavy metal contamination and antibiotics present in various concentrations in the sewage sludge [60,61]. Certain drugs such as analgesics and non-steroidal anti-inflammatory drugs (NSAIDs) contain nearly 100 compounds that have been observed in sewage sludge. Heavy metals and antibiotics together can contribute to the emergence of antibiotic resistance to these contaminants. The toxic effects of these compounds can reduce the soil microbiota, thus lowering the soil quality, making it unfit for the growth of food crops and other organisms [62]. Studies on NSAIDs in sewage sludge have been reported to be toxic to different species such as fishes in the water, arthropods, and earthworms in the soil [63]. In plants, NSAIDs are bioaccumulated, transported, and transformed into the tissues of the crops. In particular, diclofenac, paracetamol, and ibuprofen have been reported to exert cytotoxic and genotoxic effects on *Zea mays*. Ibuprofen has been proven to induce phytotoxic effects in the roots of *Lactuca sativa* during organogenesis [61,64].

Antibiotic Resistance

Because of the growth of antibiotic-resistant bacteria [12,15] and antibiotic-resistant genes, the availability of antibiotics is of great concern. They are used extensively in humans and veterinary medicines to build resistance to microbial infections. These are then excreted after a few days of consumption [65]. Despite the number of antibiotics existing, organisms mainly develop antibiotic resistance due to spontaneous mutations within their DNA structure. As antibiotics eliminate all vulnerable bacteria, the ones left are resistant to the treatments and can thrive and transmit resistance throughout the ecosystem. Most bacteria are likely to evolve, soon becoming entirely resistant to most antibiotics currently used and posing increased danger to humans and the environment [66,67].

#### 2.2.2. Pathophysiology Involved in Phthalate Exposure

Phthalates have been persistently used in the manufacture of plastics. Due to its non-covalently bound nature, plastics can easily leach away and contaminate the environment. It can lead to bioaccumulation in plants and other food crops due to their phthalate-contaminated soil and water [68]. Phthalates can easily sneak into animals and the human body due to the widespread use of plastics and their improper disposal, which creates reproductive toxicity in the exposed organisms, depending on the dose and duration of the exposure. Numerous research works have reported that phthalates are associated with developmental and reproductive defects in offspring—both in males and females [69]. Active phthalate compounds generally target the endocrine system and create reproductive anomalies. Increased exposure to phthalate can induce disturbances in the pro and antioxidant levels in the cells of the testes, causing impairment in the testicular function, which can then lead to apoptosis. The disruption of antioxidants in those cells allows ROS production to increase the oxidative stress attributed to DNA and tissue damage, mutagenesis, and even cell death [70,71]. Studies have observed that phthalate-induced toxicity in females has less significant effects than that in males. When adult female rats were exposed to Di-2-Ethylhexyl phthalate (DEHP), they had advanced puberty, altered ovulatory cycles, prolonged estrous cycles, and delayed conception [72]. Certain studies have also reported that phthalate-exposed females had decreased oocyte penetration and implantation, delayed fertilization, and sometimes lost implanted embryos and embryogenesis [73]. In males, apoptosis and oxidative stress together have led to the impairment of spermatogenesis, hampered sperm acrosome activity, and a decline in semen level [74,75]. Several pathways are responsible for phthalate toxicity. However, they mainly work as endocrine disruptors (Eds) by inhibiting reproductive enzymes, lowering the Leydig cell function, and impeding anterior pituitary and testicular hormones [75]. All these reactions inhibit the testosterone synthesis that disrupts the Sertoli cell interaction with germ cells, thus causing a disturbance in the reproductive tract; hence, the destruction of spermatocytes occurs, leading to a decrease in the sperm count and its motility in males [70,76].

#### 2.2.3. Pathophysiology Involved in the Exposure of Marine Plastic Debris

The fate of microplastic debris being found in the marine environment originated mainly from wastewater treatment plants and sewage systems that cause the deposition of plastic debris in the seas and oceans [77]. Recently, studies have reported that this plastic debris can serve as a vector in taking up hazardous chemicals by the interaction of those chemicals with polymers and their subsequent translocation to different trophic levels across the food chain. Adsorbed chemicals and plastics, when they have reached the guts of the marine organisms, disrupt the endocrine system using endocrine-disrupting chemicals (EDCs) and cause an imbalance in the hormonal levels of the organism, either serving as an estrogen agonist in females or as an androgen agonist in males [78,79]. It also affects organisms such as crustaceans and other aquatic fishes, creating a disturbance in their hormonal regulation that can lead to metabolic changes by upsetting their biochemical pathways [30]. Ingestion of these contaminants occurs when this plastic debris is suspended in the water. Fishes can take in water through which various plastic debris, alone or in a mixture, can be ingested, depending on various factors. Some predators may falsely recognize these contaminants as prey due to their attractive colors. For example, *Aethia psittacula,* which feeds typically on light brown-colored crustaceans, can erroneously feed on dark-colored plastic particles. Other predators feed on jellyfish that might have ingested radiant or translucent plastics [68,80].

### 2.3. Circulation, Bioaccumulation, and Uptake

#### 2.3.1. Circulation

Circulation includes the transport of contaminants, from the place of origin to several other sources, through different pathways, depending on the type of the contaminant. Some contaminants mainly accumulate in environmental areas such as soil, urban lands, humans, animals, and plants [81]. Specific contaminants and metals released by incineration can travel up to 10 km and significantly contribute to the local pollutants. However, other contaminants that are obstinately present in the environment, either alone or in mixtures, are called persistent air pollutants [82]. These pollutants are highly resistant to physical, chemical, and biological degradation stress, causing adverse effects on the biota. They can even travel more than a hundred kilometers without changing their toxicodynamics. Some substances can be directly deposited onto the surface of the soil vegetation and in water bodies, where it comes into contact with humans and animals, causing a series of complex reactions [83]. Persistent air pollutants are highly toxic as they can be easily carried by air and cannot be controlled within a particular scale. They include dioxins, mercury, chlordane, DDT, and furans that can travel beyond countries from a small primary source [84]. They also include semi-volatile organic compounds (SVCs), high-vapor pressure metallic compounds, and persistent organic pollutants (POPs) that are highly resistant to the degradation process and can stay thriving for many years. Their significant vapor pressure allows them to emit their primary source and undergo continuous circulation in the biosphere [85,86].

#### 2.3.2. Bioaccumulation

The pollutant is deposited onto the soil by various soil transporters such as earthworms, ants, rats, microbes, and other organisms that burrow in the soil. Some contaminants are washed away horizontally, leaching out to the nearby surface waters, or are blown by the wind and disperse in the air [83]. Various other soil-contaminating POPs can be transported by erosion or volatilization and can move deep into the soil and get deposited; this is due to physical, chemical, and biological degradation processes such as leaching, diffusion, photolysis, tectonic plate movements, and microbial resilience [87]. They undergo massive changes in their structure, which may cause them to dissipate or interact with other pollutants, thus forming a new combination called newly emerging pollutants (NEPs) with enhanced toxicokinetics and toxicodynamics [88]. They can further penetrate the soil from where they can reach animals, humans, and primarily plants, bioaccumulating in the terrestrial biota [89,90].

#### 2.3.3. Uptake

Plants have a symbiotic relationship with both soil and air. They take up the contaminants directly from both sources via deposition or foliar feed, where the plant leaves take up the contaminant vapors. Many inorganic chemicals and heavy metals are mostly taken up by the hugely bioaccumulated roots in the soil [91,92]. Translocation of organic and inorganic chemicals occurs through the soil’s roots when water absorption occurs through osmosis. Contaminants exist in a dissolved state in water and are sorbed as suspended particles in the soil. When these contaminant bioaccumulated plants are consumed, they can be transported easily to humans and animals [93]. The consuming humans/animals, when they die/degrade, re-release these contaminants to the soil, thus contaminating the biosphere and continuing the cycle. However, some plants have developed resistance to certain heavy metals. Some plants naturally have this ability to hyper-accumulate metals in higher quantities. This uptake, bioaccumulation, and bioprocessing ability of plants can be exploited positively to clean up the environment [94,95].

### 2.4. Other Trending Contaminants and Their Complex Mixtures

#### 2.4.1. Triethylene Glycol Dimethacrylate (TEGDMA)

TEGDMA is used as a monomer to reduce the viscosity of resin-based dental supplies. The hydrophilic and lipophilic nature of TEGDMA allows them to diffuse into various biological environments, induce cytotoxicity, and create alterations in the cell cycle and protein expression [96]. TEGDMA, in millimolar concentrations, can diffuse into the pulp space through direct or indirect pulp capping made of polymerized resin composites [97]. In various studies, this has been proven to be cytotoxic and a cause of apoptosis, necrosis, and the inflammation of pulp tissue. TEGDMA can be quickly released by the polymerized dental resins into the oral cavity, pulp tissues, and neighboring cells [96]. It can effectively penetrate the cell membrane and reach the cytosol of those cells. TEGDMA induces cytotoxicity in dental pulp cells and suppresses cell growth in various mammalian cells in a dose-dependent manner, playing a pivotal role in cell cycle alteration [98,99]. TEGDMA exposure at concentrations of 1–2.5 mM/L and 5 mM/L can cause G2/M cell cycle arrest and late S phase arrest, respectively. The molecular mechanism of cell cycle alteration mainly involves cyclins and cyclin-dependent kinase (CDK) [100]. Upon treatment with 2.5–5 mM/L TEGDMA, a slight decrease in the cdc 2, cyclin B1, and cdc 25C mRNA expression of dental pulp cells was observed in protein expression. Additionally, TEGDMA at 2.5 mM/L elevated the p21 expression to inhibit all cyclin/CDK complexes [101]. It may suppress the G2 phase progression and prevent M phase entry. The concurrent effect also activates the p21, GADD45, and 14-3-3σ protein transcription, by which the cyclin B/cdc2 complex is inactivated. Thus, cdc 25C is suppressed, and the elevation of p21 is observed in TEGDMA-exposed human dental pulp cells [102]. TEGDMA-exposed dental pulp cells have been detected to undergo apoptosis and necrosis, which can be correlated with MAPK activation and cause morphological changes, thus confirming the cell cycle aberration. Several cytokines, chemokines, and prostaglandins have been reported to be generated and in which COX-2 is responsible for the enhanced production of prostaglandins in response to pulpal inflammatory stimuli. TEGDMA-exposed cells were found to have increased COX-2 expression at a concentration of 2.5 mM/L, sequentially increasing the level of PGE2 and PGF2α in dental pulp cells. Furthermore, it induces pro-inflammatory mediators such as TNF-α, IL-6, and IL-10, which also induce COX-2 expression in response to inflammation [103].

#### 2.4.2. Urethane Dimethacrylate (UDMA)

UDMA is a dental resin monomer used to synthesize dental composites for restorative dentistry. UDMA leaches from the polymeric resin composites and enters the peripheral tissues, dental pulp cells, and oral cavity. Any excess or leaky UDMA can cause cytotoxicity, genotoxicity, or apoptosis in human pulp cells [103]. There has been sufficient evidence showing the cytotoxicity and DNA damage induced by UDMA even at a concentration of 1 mM. UDMA, at concentrations of 10 and 100 µM, induce apoptosis and necrosis in macrophages, respectively [104]. Chang et al. (2020) showed a concentration-dependent increase that induced caspase-3, 8, and 9 activities in UDMA-exposed RAW264.7 macrophages. Caspase-induced apoptosis that occurs by mitochondrial dysfunction through ROS generation acts as an initiator of all other effects associated with UDMA-induced cytotoxicity caused by depolarization and is an early sign of UDMA-mediated toxicity [99,105].

#### 2.4.3. Diclofenac

The primary source of diclofenac, an anthropogenic pollutant, is the drug industry as well as human and veterinary usage [106]. Diclofenac usually ends up in the wastewater or the soil through landfills used by both sources. Diclofenac is significantly detected in water bodies such as treated wastewaters, lakes, streams, rivers, and even in freshwater sources in µg L^−1^ concentrations; it has been reported to cause cytological alterations, delayed hatching, and renal lesions in aquatic organisms at various concentrations ranging from 1 to 2000 µg L^−1^ [107]. There are three diclofenac metabolites, namely 3′-hydroxydiclofenac, 4′-hydroxydiclofenac, and 5′-hydroxydiclofenac. These are present in wastewater treatment plants and can effectively interact and result in a combination that may create another potential emerging contaminant [108,109]. This might be possible due to the hydroxyl group in the metabolites that can enhance the interaction with metals through π–π interactions. This might be the case for the hydroxyl group alone, and several other activist groups such as carboxyl groups in the metabolites of diclofenac can interact with other emerging contaminants such as pesticides surfactants. Recently, the synergistic effect of diclofenac with several other emerging contaminants suggests that the toxicological effects of any new combinations cannot be overruled [110]. A few investigations suspect that the metabolites of diclofenac are potentially more toxic than the parent compound and have an enhanced ability to interact with other organic and inorganic contaminants, metals, and even with their metabolites when present in a complex environment [111,112].

#### 2.4.4. Pharmaceuticals

Due to high consumption, the presence of pharmaceuticals in the environment and water has increased. Because of their endurance and potential for harm to the aquatic ecosystem, these biologically active compounds are classified as emerging contaminants. Some of the main components of pharmaceutical waste are antibiotics, anti-depressant drugs, chemotherapy products, and hormones. They impair water quality and negatively influence the ecosystem and human health even in low quantities [113,114].

Antibiotics

Antibiotics are frequently regarded as “pseudo-persistent compounds” due to their continuous introduction and presence in the environment. Antibiotics are of concern because they are designed to kill microorganisms. As a result, they will impede the activity of beneficial microbes in the environment that contribute to various needs of the ecosystem [115]. Furthermore, the microbial community living in wastewater develops resistant mechanisms faster than the rest of the microbial world because of constant antibiotic exposure. Antibiotic pollution has been noted to disrupt aquatic environments, inhibit the ecosystem’s normal functions, and affect human health. Long-term exposure to antibiotics has been mentioned to lead to chronic conditions such as diabetes and obesity [116,117].

Analgesics

These comprise a group of drugs used to relieve pain and inflammation in the body, also known as painkillers. It is a heterogeneous group composed of aspirin, ibuprofen, paracetamol, ketoprofen, diclofenac, mefenamic acid, and many others. These drugs are poorly degraded and removed in water treatment plants, and they have exhibited characteristics of contamination and toxicity in a wide range of aquatic animals [118]. Paracetamol is the most commonly detected drug in water, and the chlorination of this drug has led to its transformation into a toxicant. In the case of ibuprofen, more than 15% of the drug is excreted and 26% as a metabolite; it has been found to be highly toxic to aquatic organisms than ibuprofen itself. A study by [119] found concentrations of these drugs from 2 ng L^−1^ to 18 μg L^−1^ in the water. These xenobiotics in the environment and water bodies pose a substantial risk to human health and safety due to the lack of knowledge of long-term exposure and consequences [120,121].

Lipid Regulators, Beta-Blockers, Psychiatric Drugs

Lipid regulators are substances involved in controlling blood cholesterol and triglyceride levels [122]. Beta-blockers include atenolol, sotalol, and metoprolol and are known to inhibit the beta-adrenergic action of CNS receptors, leading to the opening of the blood vessels and the anti-hypertensive effect of bronchi in the lungs after the administration of these drugs. Psychiatric drugs include anti-depressants and psycho-stimulants. They are mainly known to influence the function/release of these neurotransmitters [123,124]. Wastewater has been the most affected by these types of drugs. Existing treatment plants cannot remove these drugs precisely, so the drugs can transform into ecotoxicological substances and affect a vast number of organisms [125].

## 3. In Vitro/In Vivo Studies and Biomarkers to Understand the Combined Toxic Effects of the Contaminants

### 3.1. Biota

Biomarker responses in terrestrial species can be monitored in several ways to provide a qualitative analysis of how a particular contaminant jeopardizes the health of its surrounding environment. Extensive studies on biomarkers have resulted in a clear improvement in air/water quality and waste minimization with bioassays on fauna and flora.

#### 3.1.1. Fauna

Fauna includes all forms of animal lives living in a specific region at a specific time. Widespread contamination affects or bioaccumulates or induces changes in the animals exposed. Plastic debris is highly prevalent in marine organisms. When ingested, they accumulate this debris in their bodies [126]. When assessed, seabirds have been found to contain elevated amounts of persistent organic pollutants (POPs) and Polychlorinated Biphenyls (PCBs). Because the co-contaminant can attach to the microplastics, a study was conducted to assess co-contaminant bioavailability using gene expression in zebrafishes at the larval stage. A study was conducted on *Oreochromis niloticus*, a freshwater tilapia, assessing the cellular changes due to persistent pollutants [126,127].

Response to the accumulation of heavy metals (Cr, Cd, Cu, Zn, Mn, Fe) and co-contaminants enhanced lipid peroxidation in the muscle, gills, and livers, indicating the onset of oxidative stress in the marine organisms. These lipid peroxidases further destroy the membrane lipids by forming unsaturated fatty acids [128]. The free radicals that are generated have been observed to initiate genotoxic effects. ROS can also decrease superoxide dismutase and glutathione peroxidase (GPx) activities in the liver and the gills of *O. niloticus* [129]. Similar effects were observed in the liver, gills, and heart of *Gasterosteus aculeatus, Brycon amazonicus,* and *Channa punctatus.* Glutathione S-Transferase (GST) activity is also reduced in the lungs and gills of tilapia, indicating a response to the presence of polyaromatic hydrocarbons (PAHs). Metallothionein also seemed to be overexpressed and was found in elevated levels in gills of tilapia, indicating heavy metal exposure in them [128,130].

#### 3.1.2. Flora

Environmental pollutants are highly deposited in soils, absorbed by the plant life—the so-called flora and crops cultivated on them—and supplemented with wastewater irrigation. The heavy metal concentrations in the soil and water are taken up and accumulated considerably depending on the amount of contamination present. This can be monitored by assessing the exposed plants as biomarkers. Many crops such as *Ocimum basilicum, Raphanus sativus*, *Coriandrum sativum*, *Allium ampelpprasum*, and *Beta vulgaris* that have grown on contaminated soil have undergone cellular changes and experienced stunted growth and decreased crop productivity [131,132,133]. *Amaranthus caudatus* takes up Cd at higher concentrations (1.5 µg/g) as well as other metals such as Cr, Pb, and Zn. One of the significant biomarkers is the production of ROS, causing deleterious damage to the DNA, protein, and lipids [134]. However, plants are equipped with a series of antioxidants that enhances their defense mechanism. These enzymes potentially help in the detoxification of H_2_O_2_, mainly in the region of chloroplasts. However strong the plant system may be, metal accumulation at higher concentrations could damage the antioxidant itself [134]. A similar mechanism has been noted to be prevalent in many other plants; in particular, *Avicennia marina* has been investigated for its antioxidative stress-responsive genes. The authors identified a strong upregulation of PPOA in the leaves and seedlings when these were exposed to oil contamination. In *Arabidopsis thaliana*, the upregulation of the NAP2 gene has been observed when exposed to phenanthrene and oil spills [135,136].

### 3.2. Haematological and Immunological Factors

Mycotoxins such as deoxynivalenol and zearalenone have been observed to cause hematological and immunological changes, altering the gene expression involving inflammatory cytokines and chemokines [137]. These food contaminants drastically impacted the feed intake pattern of mycotoxin-exposed pigs and increased IgG and IgM, thus damaging their vital organs and suppressing antioxidants and serotonin levels. They were also expected to be involved in the onset of infections that trigger inflammatory cytokines and create lesions in the liver and kidney. Another study assessed the effects of trichothecene T-2 mycotoxin contamination in rainbow trout [138]. The results of this study indicated a dose-dependent relationship between mycotoxin and the immune response. A decrease in plasma count and increased leukocyte count were observed with elevated T-2 toxin concentration. It also downregulated non-specific immunity in the test groups compared to the control. Another study evaluated an olive leaf extract’s ability to reduce ammonia toxicity in common carps. The fishes were given 1–10 g/kg of the extract, with 0.5 mg/L ammonia exposure for 60 days, which resulted in free radicals and malondialdehyde levels in the plasma and decreased human corpuscular hemoglobin [139,140,141].

### 3.3. Histological Factors

A study examined the effects of various mixtures of anti-depressants in adult fish, which highly affected the fish’s liver and muscles. The authors observed relatively less proliferation of hepatocytes, which enhanced mitochondrial oxidative stress and caused hypoxia, thus indicating liver damage [142]. These were obtained from the histopathological analysis of the target organs’ tissue samples through biopsies. Fishes at the larval stage experienced delayed differentiation and lowered morphogenesis on combined anti-depressant exposure [143]. Significantly, various studies have highlighted the level of contamination in marine populations leading to carcinogenesis. A histological study that involved liver tissue containing a cluster of cells indicated a lesion at the site of PAH contamination. They also contained elevated melanomacrophage centers at the PAH-contaminated site, which was identified to be a histological indicator of xenobiotic transformation [144,145].

### 3.4. Biochemical Factors

Parameters such as free amino acids, sugars, ascorbic acid, protein, DNA, RNA, chlorophyll, superoxide dismutase, catalase, and peroxidase are the biochemical factors found to be present in plants and a few animals, including humans [146]. These factors can be altered when they encounter a new or mixed pollutant. The changes or variations in the functioning of these parameters can be used as bioindicators in identifying and quantifying environmental contaminants. The biochemical changes in selected plant species, namely *Azadirachta indica, Mangifera indica, Cassia fistula*, and *Delonix regia,* from significant urbanized areas in a similar study reported that the air pollution caused by anthropogenic activities emitted greenhouse gases (GHGs) and other toxicants in the air [147] that adversely affected the chlorophyll, pH, moisture content, and antioxidants in crops such as *Oryza sativa* [148,149]. However, their toxicity increases when GHGs such as SO_2_ and NO_2_ combine to make a toxic mixture. These pollutants also increased the cell permeability and penetrated the cells of the plants, forming minute pores that accumulate in them. As a result, the stomata were closed to reduce the loss of water. Particulate penetration hampered various metabolic processes such as photosynthesis, where CO_2_ accumulates in plants due to stomata closure [150]. An increase in antioxidants and free radical scavengers in response to the production of ROS and reactive sulfites during photo-oxidation, the depletion of sugar contents due to the reaction of sulfites with carbohydrates, and the denaturation of proteins by breaking down the existing form and reducing them has been observed in *Cassia fistula* when exposed to heavy and moderate air pollution. These biochemical alterations have changed the metabolic processing of a plant that serves as a latent biomarker in response to environmental pollution (Table 1) [145,151].

**Table 1:** This table provides a complete overview of the categories of biomarkers, contaminants’ combined effects, mechanism, and severity in various organisms.

Category	Biomarkers	Contaminant Type	Concentration Exposed	Organisms Exposed	Organs Affected	Pathophysiology	Endpoint/Effect Evaluated	Severity	References
Fauna	Bioaccumulation of POPs, PCBs	Plastic debris	5 mg/L	Seabirds, Zebrafishes (larval stage)	Livers, gills, and lungs	Hyperaccumulation of plastic debris in elevated levels	Changes in gene expression, Teratogenic	Moderate	[126,127,128]
	ROS, Overexpression of metallothionein	Heavy metals (Cr, Cd, Cu, Zn, Mn, Fe)	0.2–0.6 mg/kg	*Oreochromis niloticus, Gasterosteus aculeatus, Brycon amazonicus,* and *Channa punctatus*	Heart muscle, gills, lungs, and livers	Free radical generation, Oxidative stress, Lipid peroxidation	Genotoxic effects, Forms unsaturated fatty acids	Low	[152,153,154]
Flora	Decrease in chlorophyll and carotenoid content, bioaccumulation of heavy metals	Heavy metals (Ni, Cu, Cr, Pb, and Zn), pharmaceuticals, and personal care products	40–50 mg/kg	*Ocimum basilicum, Raphanus sativus, Coriandrum sativum, Allium ampelpprasum,* and *Beta vulgaris*	Leaves, roots, and stem	Lipid peroxidation, increased antioxidant, Cellular changes	Stunted growth and decreased crop productivity	Moderate	[133,134,155]
	ROS, NOS	Cd along with other metals	1.5 µg/g	*Amaranthus caudatus*	Roots and stem	Production of ROS, NOS	Damage to the DNA, protein, and lipids	Moderate	[155,156]
	Upregulation of PPOA, upregulation of the NAP2 gene	Oil contamination (phenanthrene and oil spills)	<10% (*w*/*w*)	*Avicennia marina, Arabidopsis thaliana*	Leaves and seedlings	Upregulation of antioxidative stress-responsive genes	Alteration in gene expression	Low	[135,136]
Hematological and immunological Factors	Increased IgG and IgM	Mycotoxins	4700–11,400 µg/kg	Rainbow trout, pigs	Liver and kidney	Decrease in plasma count and increased leukocyte count, upregulation of inflammatory cytokines and chemokines	Alteration in the gene expression, decreased non-specific immunity	Low	[137,138,139]
	ROS, Malondialdehyde	Ammonia	0.5–1.8 mg/L	*Cyprinus carpio* (Common carps)	Muscle and gills	Generation of free radicals, upregulation of antioxidants, and apoptosis-related genes	Decreased the human corpuscular hemoglobin, convulsions, coma, and death	High	[140,141]
Histological Factors	ROS	Mixtures of anti-depressants	~25 µg/L	Larval and adult fishes	Liver and muscles	Mitochondrial oxidative stress causing hypoxia	Delayed differentiation and lowered morphogenesis (in the larval stage), the lesser proliferation of hepatocytes, liver damage (in adults)	High	[143,144,146]
	Elevated melanomacrophage centers	PAH	0.1–8 g/L of crude oil	Adult Fishes	Liver tissue	Increased cell growth, megalocytic hepatosis, and vacuolations in the liver tissue	Hepatic lesions, Carcinogenesis	High	[144,157]
Biochemical Factors	Bioaccumulation, decreased chlorophyll and carotenoids	Greenhouse gases (GHGs)	-	*Azadirachta indica, Mangifera indica, Cassia fistula,* and *Delonix regia*	Roots and leaves	Increased cell permeability, penetration, forming minute pores, bioaccumulation, closure of stomata	Hampered metabolic processes, CO_2_ accumulates in the plants due to the closure of stomata	Low	[158,159]
	Malondialdehyde, increased antioxidants	Combination of SO_2_ and NO_2_	SO_2_—4–8 ppb and NO_2_—39.9 ppb	*Oryza sativa*	Shoot	Particulate penetration, Lipid peroxidation	Changes in pH, moisture content, and antioxidants, distortion of leaves, and reduced yield	Moderate	[151,160]
	ROS, depletion of sugar, increase in antioxidants	Heavy metal and moderate air pollution	-	*Cassia fistula*	Seedling	The reaction of sulfites with carbohydrates, denaturation of proteins	Biochemical alterations in the metabolic processing of the plants	Low	[149]

### 3.5. Case Studies

UDMA individually or in combination with TEGDMA has been reported to induce cytotoxicity in mouse fibroblasts [161]. The outcome was slightly higher than the effect of TEGDMA individually. UDMA exhibited a genotoxic effect at 1 mM, whereas TEGDMA exhibited the same effect at 5 mM, which shows that each of the monomers has its specificity acting at a specific concentration and under different time conditions [99,162].

A study examined the combined effect of eight heavy metals and metalloids [104] and found that these caused lipid peroxidations in the bone marrow and spleen cells in a dose-dependent manner. They also reported inducing micronuclei formation and sister chromatid exchange, causing cytogenetic damage to the bone marrow cells. It has been speculated that oxidative stress-induced DNA damage can also interfere with the DNA repair mechanism. Thus, the production of ROS and nitric oxide synthases (NOS) can be a predictive biomarker in estimating metal toxicity [99,105]. Another study of a heavy metal cocktail of eight metals in male albino rats has been found to suppress the humoral and cell-mediated immune response, which could cause anemia due to immunotoxicity [104,163].

A study involving albino rats found the possibility of the metal mixture crossing the blood–brain barrier and potentially reaching the hippocampus, frontal cortex, and other regions of the brain, which then inhibits the N-methyl-D-aspartate (NMDA), acetylcholinesterase (AchE), glutamic acid decarboxylase (GAD), GPx, and GSH cellular elements and initiates mitogen-activated protein kinases (MAPK) and the extracellular signal-regulated kinase (ERK) pathway; this can lead to neuronal cell death, cognitive dysfunction, and cause various neurological diseases such as Alzheimer’s and Parkinson’s disease, Huntington disease, autism, multiple sclerosis, and Wilson disease. Researchers estimate a high chance of cancer occurring due to the dose-response connection with the heavy metals and their mixture [161,164].

## 4. Techniques to Overcome Mixed Contaminant Toxicity

Due to various anthropogenic activities, different micropollutants are being released into the ecosystem, causing several adverse health effects on humans and other living organisms [165]. The tremendous increase in these pollutants over the last few years has necessitated the demand for more cost-effective ways of controlling pollution. Several techniques have been introduced to control the contaminant levels in aquatic, soil, and air environments (Table 2).

### 4.1. Containment-Immobilization Technologies

#### 4.1.1. Containment Technologies

Containment is a prevalent solution used to contain physically and hydraulically contaminated areas. This method is used to avoid the leaking or leaching of contaminants into the surroundings. There are two types of containment systems, namely passive and active forms. There is no need for an ongoing energy input in the passive containment system. In contrast, continuous energy input is required in the active system. Containment can be effectively established in unconsolidated soil [166,167].

#### 4.1.2. Immobilization Technologies

Immobilization methods most commonly involve organic and inorganic additions that will help speed up the reduction of metal mobility and toxicity in soil. The primary function of this technology is to change the soil metals into their stable phase through various processes such as sorption and precipitation. Immobilization techniques are applied in heavy metal-contaminated areas [168]. Several studies have reported using clay to immobilize heavy metal-contaminated soil. Clay immobilizes the metal particles through an adsorption mechanism and is retained on the surface due to its increased surface areas. Another technique used for immobilization is the introduction of binders. Binder components such as calcium silicate hydrates attract heavy metals and are fixed on these components. They immobilize the heavy metals and have other beneficial effects such as reducing the permeability of the soil. This prevents the heavy metals from migrating and contaminating nearby areas. It also increases the pH of the soil, resulting in reduced soil acidity. The mobility of heavy metals is then reduced due to this decreased acidity [169,170].

### 4.2. In Situ Methods

In situ methods help treat the subsurface contaminants. Unlike ex situ techniques, it does not require moving the soil from its original place. There are mainly three in situ techniques: chemical, physical, and thermal. As these techniques could not produce efficient results due to the shifting of this pollution into the air, other techniques were introduced. Biological treatments use various microorganisms and help to control the shifting of pollution and its degradation [171].

Some commonly used biological in situ techniques are bioaugmentation, bioventing, bio slurping, phytoremediation, phytoremediation [2,172], and sparging. Because there are no excavation or transportation costs, in situ remediation is more economical, although it is less controlled. Microbial bioremediation occurs in contaminated sites under aerobic and anaerobic circumstances and as intrinsic and accelerated biodegradation. The type of organisms, pollutants, and the geological and chemical conditions at the polluted site influence whether microorganisms can successfully eliminate artificial toxins in the subsurface. Biostimulation and bioaugmentation are two critical techniques for improving bioremediation, assuming that environmental parameters that impact bioremediation performance are kept at appropriate levels [171,173].

#### 4.2.1. Bioaugmentation Technique

The introduction of microorganisms that have the potential to biodegrade resistive compounds in a contaminated environment is known as bioaugmentation. Bioaugmentation is most often used to restart activated sludge bioreactors in treating wastewater. Quinolines and pyridines are common chemical compounds in dyes and paints [174]. Due to their low biodegradability, they persist in the environment for a long time. Their carcinogenic activity makes it dangerous for humans. Several studies have been conducted, and it has been found that the biodegradation of quinoline can be achieved with *Burkholderia pickettii.* They can also be bio augmented with *Paracoccu* sp. and *Pseudomonas* sp. Synthetic dye such as azo dye can be bio augmented using *Shewanella* sp. [175,176].

During the coking process in the steel industry, cyanide is released, which is one of the most dangerous substances generated by coal [177]; it is eventually accumulated in the environment. It can be bio-augmented using *Cryptococcus humicolus*, a cyanide-degrading yeast. *Acinetobacter* sp. and *Sphingomonas* sp. have been discovered as bacteria capable of digesting nicotine, another contaminant released into the environment [178]. PAHs are another group of pollutants that can be bio-augmented using strains of *Streptomyces* sp. One of the advantages of bioaugmentation is that these treatments can be designed for a particular pollutant prevalent in the environment. In this way, the pollutants present in increased concentrations are addressed as well as the increase in emerging pollutants [174,176].

#### 4.2.2. Bioventing Technique

The only in situ remediation strategy that enables the treatment of unsaturated soil is bioventing. The fundamental basis of bioventing is to feed with air and nourish contaminated soil through specially constructed wells in order to promote the indigenous microorganisms [179]. The bioventing technique uses reduced airflow rates to provide the oxygen needed for biodegradation and to reduce the volatilization and emission of contaminants into the atmosphere. Because vapor migration via active biological membranes is generally slow, this approach aids in the degradation of adsorbed fuel residuals. Research shows that in just 7 months, phenanthrene-contaminated soil had 93% of the contaminants removed by bioventing. With longer air injection intervals and lower air injection rates, the bioventing technology may prove to be cost-effective in eliminating diesel from clay soil [180,181].

#### 4.2.3. Biosparging

Biosparging is the process of injecting air or oxygen as well as nutrients, if necessary, into a saturated zone to encourage microbial activity. This technology targets chemical substances that can be biodegraded under aerobic conditions and treat soluble and residual contaminants in the saturated zone. A biosparging system aims to increase pollutant biodegradation while minimizing volatile and semi-volatile organic compound volatilization [182]. Biosparging has been widely employed in treating petroleum-contaminated aquifers, particularly diesel and kerosene. Benzene, ethylbenzene, toluene, and xylene-contaminated groundwater plumes have been biosparged, transitioning from anaerobic to aerobic conditions. The increased reduction of contaminants in these chemicals indicate that sparging could be used as a bioremediation process for areas with contaminated ground water [183,184].

### 4.3. Ex Situ Methods

Ex situ methods entail extracting pollutants from polluted places and delivering them to a treatment facility elsewhere. The expense of treatment, the degree of contamination, the type of contaminant, the geographical position, and the nature of the contaminated site are all factors to consider in ex situ methods. The significant advantage of ex situ treatment is that it usually takes less time and provides more assurance regarding treatment homogeneity. Despite this, ex situ methods require an excavation of the soil and its treatment, making it less cost-effective [185].

#### 4.3.1. Biopiling

Biopiling is a bioremediation technique that involves stacking toxic soil above ground, followed by nutrient enrichment and, in some cases, aeration in order to improve bioremediation by essentially boosting microbial activity [186]. The usage of a biopile in polluted areas can assist in minimizing the volatilization of low molecular weight contaminants. In this process, the contaminated soil is heaped up to a few feet of depth, and then the aeration pipe is placed. Afterward, the next batch of polluted soil is introduced. This method is continued until the pile level is achieved. The soil is generally combined with bulking agents such as straw to boost aeration and expand the microbial population. Biopiling has been effectively utilized to remediate soil polluted with crude, diesel, and lubricating oil [187,188].

#### 4.3.2. Land farming

Land farming often employs a mix of volatilization and biodegradation to lower hydrocarbon concentrations [189]. Stimulating aerobic soil microorganisms is necessary for successful biodegradation; this is generally performed by providing nutrients and blending the soil to enhance the aeration. The excavated soil sample is distributed as a thin layer over a treatment bed coated with an impermeable layer in order to limit leaching and discharge in ex situ landfarming. In treating petroleum hydrocarbons and some less volatile, biodegradable pollutants, land farming has proven to be the most effective. Land farming is commonly employed to clean up toxic hydrocarbons such as polyaromatic hydrocarbons [190,191].

#### 4.3.3. Composting

Composting is a regulated biological process in which microbes transform organic pollutants such as PAHs into harmless, stable metabolites under aerobic and anaerobic conditions [192]. The technique has attracted greater attention in recent decades since it has demonstrated its high effectiveness in degrading various organic pollutants, including PAHs, pesticides, explosives, and chlorophenols. Some pollutants such as polycyclic aromatic hydrocarbons (PAHs) [193,194,195] have been demonstrated to degrade more quickly at a higher temperature, even at the risk of gaseous volatilization [192]. In some cases, anaerobic conditions are required to break down chlorinated compounds, followed by aerobic treatment to disintegrate partially dechlorinated compounds and other components. The reaction environment’s physical, chemical, and biological factors influence the effectiveness of composting in the bioremediation of polluted soils [196]. The microbiological accessibility of the contaminants is a significant aspect influenced by operating parameters such as mixing, moisture content, soil composition, and the features of the amending agent that must be chosen for each situation. Composting efficiency is also influenced by amendment stability, which several authors have extensively studied in the bioremediation of PAH-contaminated soil. Composting has been used to remediate soil polluted with explosives and polychloro-biphenyls (PCBs) in addition to PAH bioremediation [197,198].

#### 4.3.4. Bioreactors

Microbial bioreactors are useful for remediation because they provide a stable environment where important process factors may be regulated to enhance microbial bioremediation activity [199]. Contaminants can be added to the reactor as a dried or slurry material for treatment. Pollutants having hydrophobic characteristics, especially those attached to the soil matrix, are frequently unsuitable for microbial breakdown. Compared to degradation that occurs in in situ techniques, a wide range of microbial species can naturally degrade organic pollutants. The usage of well-developed and optimized microbial bioreactors ensures enhanced degradation rates [200,201,202].

#### 4.3.5. Precipitation Technique

Precipitation is a method for extracting chemicals from a solution by introducing reagents that enable insoluble particles to form. The chemicals create particles that settle and remove impurities from the water. This method softens water and eliminates arsenic [203], fluoride, phosphorus, ferrocyanide, and heavy metals. Fluoride removal from wastewaters by reducing it to CaF_2_ has become the most extensively utilized treatment technology. Precipitation is also used in the removal of dyes [204,205]. Dyes are non-biodegradable, and CaCO_3_ precipitation is one method for removing them from water. Research conducted recently has proved the efficiency of microbially produced calcite precipitation with its successful Pb, Zn, and Cd bioremediation. The exchangeable Pb, Zn, and Cd fractions in soil were dramatically decreased after *S. pasteurii* bioremediation [206,207].

#### 4.3.6. Microfiltration

The microfiltration technique is often used to remove coarse particles or microorganisms with sizes ranging from 0.025 to 10.0 m. This technique can remove bacteria, paint, pigment, yeast cells, and other suspended debris from water using microporous membranes with enormous pore sizes, mostly between 50 nm and 5 m [208]. A pressure of 1–5 bars is generally required in this pressure-driven process. Even when seawater is substantially contaminated and includes enormous volumes of organic material or oil, membrane filter pre-treatment for marine reverse osmosis desalination plants permits pure water to be consistently supplied into the reverse osmosis modules. Shipping accidents and discharge from offshore oil and gas rigs produce a substantial amount of pollution in the world’s waters. New pre-treatment methods such as micro-filtration might be utilized to clean up oil spills [209,210].

#### 4.3.7. Electrodialysis Technique

Electrodialysis is a method that uses ion-exchange membranes and electrical potential as a key driving force to separate substances. Electrodialysis is primarily used to desalinate brackish water in order to produce drinking water on a large scale. On the other hand, considerable research has concentrated on using this approach in the biochemistry, food processing, and pharmaceutical sectors, spanning wastewater treatment, chemical or other helpful product extraction, and hazardous substance removal [211]. The low-pressure action, low lifecycle costs, and low maintenance costs are the critical advantages of ED for liquid waste treatment. Although the electrodialysis process entails a significant initial investment, it provides a higher output that is more environmentally beneficial than other mass separation procedures. Specific reports about the use of ED in the nuclear industry have also been indicated, such as the extraction of molybdenum, carbonate, and bicarbonate ions from uranium-containing liquid waste during the uranium ore leaching process. Electrodialysis is a promising technology for treating radioactive liquid waste. It enables high desalination rates and the removal of significant amounts of radioactive compounds (Table 2) [211,212].

**Table 2:** This table summarizes the updated remediation technologies and their characteristics for controlling the contamination of each type of contaminant.

Type	Technique	Components Used	Areas Applicable	Advantage	References
Containment-Immobilization	Containment	Hydraulic and physical	Soil and groundwater	Avoids leaking or leaching of contaminant to surroundings	[213,214]
	Immobilization	Organic and inorganic additors used	Heavy metal contaminated areas	Increases Ph of the soil, making it less acidic, reduces the permeability of soil, reducing migration	[215,216]
In Situ	Bioaugmentation	Microorganisms are used	Soil, surface water, and groundwater	Can be designed for a particular pollutant prevalent in the area	[217,218]
	Bioventing	Feeds air and nourishment to contaminated soil in constructed well to produce indigenous microorganisms	Unsaturated soil	Reduces the volatilization and emission of contaminants, cost-effective	[219]
	Biosparging	Injection of air for microbial activity	Saturated zone of petroleum-contaminated aquifers	Targets chemical substances that can be biodegraded under aerobic conditions	[220]
Ex Situ	Biopiling	Stacking of soil above ground, aeration, and enrichment for microbial growth	Soil is polluted with crude, diesel, and lubricating oil	Cost-effective can restore contaminated soil in extreme conditions	[187,221]
	Land farming	Volatilization and biodegradation, contaminated soil distributed as a thin layer treatment bed with an impermeable layer	Groundwater and soil, contaminated sites with hydrocarbons such as polyaromatic hydrocarbons	Low cost and lack of equipment requirements	[222]
	Composting	Use of microbes under aerobic and anaerobic conditions, thermophilic temperatures from 54 to 65 °C typically maintained	Soil is polluted with explosives and polychloro-biphenyls (PCBs) in addition to PAH bioremediation	High effectiveness in degrading a variety of organic pollutants	[192,223]
	Bioreactors	Temperature, pH, moisture, pollutant mix and concentration, and macronutrient content	Contaminants added to the reactor as a dried or slurry material for treatment	Cost-effective	[224]
	Precipitation	Chemicals are used to create particles that settle and remove impurities	Water	Technologically simple and economically advantageous	[225]
	Microfiltration	Microporous membranes with enormous pore sizes, mostly between 50 nm and 5 m	Remove coarse particles or microorganisms with sizes ranging from 0.025 to 10.0 m	Cleans up oil spills, good separation efficiency	[210,226]
	Electrodialysis	Ion-exchange membranes and an electrical potential	Desalinate brackish water for the production of drinking water	Due to high desalination rates and significant removal of radioactive compounds, it can treat radioactive liquid waste	[227]

### 4.4. Microbial Degradation

Biodegradation using artificially chosen microbial communities can be a quick, cost-effective, environmentally benign, and socially acceptable technique for the removal of contaminants without previous knowledge of the species involved or of the degradation routes [228]. Several investigations have recently revealed that various microbes and enzymes can decompose artificial polymers. The ability of numerous microorganisms and enzymes to degrade plastics such as PE, PS, PP, PVC, PUR, and PET and the microbial metabolic routes of plastic depolymerization by-products have been demonstrated in several studies. Several studies have also reported that hydrocarbon pollutants can be degraded by various indigenous microbes found in water and soil (Table 3) [229,230].

#### 4.4.1. Bacteria

Several bacteria have been discovered to feed mainly on hydrocarbons and are commonly known as hydrocarbon-degrading bacteria. Nitrate-reducing bacterial strains such as *Pseudomonas* sp. and *Brevibacillus* sp. were obtained from petroleum-contaminated soil that biodegrades hydrocarbons under aerobic and anaerobic conditions [231]. PCB biotransformation is possible with both anaerobic and aerobic microorganisms. Anaerobic bacteria dehalogenate higher chlorinated PCBs by reductive dehalogenation, while aerobic bacteria oxidize lower-level chlorinated biphenyls. The extensive use of bacteria to degrade oil spills in the ocean has also been reported in several studies [232]. *Pseudomonas fluorescens*, *Pseudomonas aeruginosa*, *Bacillus subtilis*, *Bacillus* sp., *Alcaligenes* sp., *Acinetobacter lwoffi*, *Flavobacterium* sp., *Micrococcus roseus*, and *Corynebacterium* sp. were among the nine bacterial strains identified from the contaminated stream that could breakdown crude oil [233,234]. Actinobacteria contribute significantly to the breakdown and to humus production, which helps to recycle refractory biomaterials. Studies have shown that the members of the phylum Actinobacteria play an essential role in the polylactic acid (PLA) and rubber degradation process [23,24].

#### 4.4.2. Fungi

Several studies have reported that fungi can break down the PAH with high molecular weight. Due to their similarities with lignin, a lengthy, aromatic family of compounds found in wood and PAHs, a wide variety of fungi have evolved effective mechanisms to target specific PAHs [235,236]. Cleaning up oil from various terrestrial and aquatic environments using mechanical or chemical methods such as booms, skimmers, or burning may be less beneficial than using fungi in bioremediation. Fungi in bioremediation are also comparatively cheap because they can be grown on a variety of inexpensive agricultural or forest wastes. Hydrocarbon degradation by yeasts and filamentous fungi has long been described, confirming that most fungal species are good hydrocarbon degraders [237,238].

#### 4.4.3. Genetically Modified Microbes (GEM)

GEMs have shown promise among bioremediation techniques for soil, wastewater, and activated sludge, with their improved biodegradation capacities for a wide variety of chemical pollutants [239]. Recent studies have produced new methods of introducing novel strains with necessary properties that would help in different bioremediation processes. They involve designing and adapting different catabolic pathways, modifying the affinity and the specificity of various catabolic enzymes (Table 3) [240].

**Table 3:** Microbial organisms used for the bioremediation process of various mixed contaminants.

Organism	Species	Contaminant	References
Bacteria	*Pseudomonas* sp. and *Brevibacillus* sp.	Hydrocarbons	[241]
	*Flavobacterium* sp.	Pentachlorophenol, Naphthalene	[242]
	*Corynebacterium* sp.	Pyrene	[243]
	*Acinetobacter lwoffi*	Phenol, Rubber	[244]
Fungi	*Phanerochaete chysosporium*, *Trametes versicolor*, *Bjerkandera adjusta*	Endocrine-disrupting chemicals, pharmaceuticals, and personal care products	[245,246]
	*T.versicolor* and *Lentinus tigrinus*	Cresolate contaminated soil	[237,247]
	*P. ostreatus*	PAHs-phenanthrene and pyrene	[248]
	*Aspergillus niger*, *A. foetidus,T. viride*, *A. sojae*, *Geotrichum candidium*, *Penicillium* sp.	Textile dye decolorization	[249,250]
	*Aspergillus, Curvularia, Acrimonium*	Heavy Metals	[251]
Genetically modified microbes	*Pseudomonas* sp. *B13*	Mono/dichlorobenzoates	[252]
	*E. coli JM109*	PCB, benzene, toluene	[253]

#### 4.4.4. Indigenous Microbes

Indigenous microorganisms are a type of inherent microbial consortia that lives in the soil and on surfaces, including those of all living things, and can biodegrade, bleach, bio compost, fix nitrogen, improve soil fertility, and produce plant growth hormones [254]. Aquatic as well as oil-bearing deep subsurface settings are home to indigenous microorganisms. According to different findings, aerobic indigenous bacteria are involved in the degradation of petroleum oils [255,256].

### 4.5. Phytoextraction

Heavy metals such as copper, cadmium, mercury, lead, arsenic, chromium, nickel, and zinc, which are resistant to degradation and dangerous to humans, have polluted the soil as a result of increased industrial activity. Many physiochemical strategies for removing metals from soil have been developed, but none of them is safe or effective. Phytoextraction, also known as phytoaccumulation, is a promising soil remediation process that may quickly remove heavy metals and cleanse the soil of toxins. Plants have a built-in system that allows them to take in and store nutrients based on their bioavailability in the soil and the needs of the plant [40,257,258]. Phytoextraction can also be defined as a type of phytoremediation during which plants extract toxic contaminants or compounds from water or soil, most often heavy metals, which will have a greater density and can be poisonous to species at low concentrations. The heavy metals that plants collect are toxic to them, and the plants being used for phytoextraction are recognized as hyperaccumulators, storing enormous levels of heavy metals within their tissues. Plants that take in lesser amounts of pollutants may also conduct phytoextraction, and because of their rapid growth rate and mass output, they can remove a significant quantity of toxins from the soil. Heavy metals may be a significant concern for any biological entity since they react with a variety of substances that are necessary for biological functions [259]. They can also break down other compounds into further reactive species, causing biological processes to be disrupted. These interactions reduce the saturation of important molecules while also producing hazardously reactive molecules such as the radicals O and OH. Since so many heavy metals seem to be chemically similar to other elements that are important to the plant’s survival, non-hyperaccumulators can also uptake some concentrations of heavy metals [257,260].

### 4.6. Phytoremediation

Contaminated soils and streams are serious human health and environmental issues that new phytoremediation technologies may help to partially alleviate. This low-cost plant-based remediation method makes use of plants’ extraordinary capacity to condense elements and chemicals from the environment while also metabolizing diverse substances in their tissues. Phytoremediation primarily targets harmful substances and organic contaminants. An understanding of the molecular and physiological processes of phytoremediation as well as biological and technical solutions for enhancing and improving phytoremediation have emerged in recent years [261]. Recently, a Monviso clone has been applied to degrade the PCBs and heavy metals in a multi-contaminated area; during this process, the rhizodegradation of PCB and the phytostabilization of HM were observed [262]. A poplar-based phytoremediation strategy was also applied in lindane-contaminated soils to remediate heavy metals by phytostabilization, which improved the overall soil quality in terms of organic and microbial activity [263].

### 4.7. Phytostabilization

The process of phytostabilization reduces the movement of pollutants into the soil by adsorption into roots, adsorption with accumulation by roots, or precipitation inside the root zone [264]. Vegetation is utilized to stabilize the migration of toxins through leaching and erosion together with soil, water, or air in order to avoid the contamination of surface and groundwater and the surrounding habitats. Plants suited for phytostabilization should have an extensive root system that allows for excellent soil colonization, contaminant metal tolerance, and the ability to immobilize pollutants in the rhizosphere [265]. Metal-tolerant species of grass, including *Agrostis capillaries* and *Festuca rubra*, are commonly used in this procedure. However, the leguminous species *Lupinus albus* has also been recommended as a viable choice for the remediation of cadmium and arsenic-contaminated soil [266,267]

### 4.8. Gomeya or Cow Dung

Cow dung helps to remove pesticides from soil and water to detoxify the environment. *Pseudomonas plecoglossicida* and *Pseudomonas aeruginosa* help to degrade cypermethrin and chlorpyrifos [268]. These bacteria from cow dung can be used for bioremediation in laboratory settings. They can also be used in pesticide-contaminated soil and water. In the bioremediation of fenvalerate-supplemented soil, activated cow dung slurry is employed as a source of microbial consortia. A mixture of cow dung poured over an oil spill in the water can soak up the oil. Bacteria found in cow dung can break down heavy crude oil into simple and harmless chemicals. This technique has been hypothesized as a solution for the preservation of marine health [269,270].

## 5. Conclusions

Due to the advancement of industrialization, a tremendous number of contaminants are being released into the environment. This has created the urgent need to find novel methods that will help understand and detect the contaminant levels and control them to an extent. Once these hazardous contaminants are released into the surroundings, they end up in living organisms, including humans, which can lead to various health complications. In designing proper control methods against these contaminants, the main factor is to understand the properties, the mode of action, the rate of uptake and bioaccumulation, and the possible effects on living organisms. The occurrence of new contaminants has led to severe challenges. The development of several biomarkers has helped to detect even the most negligible concentration of these contaminants, thus leading to new remediation technologies. Genetically modified microbes have proven to be greatly advantageous in the biodegradation of various hydrocarbons. The idea of using genetic engineering to remove heavy metals has sparked intellectual concerns. Despite these benefits, microbial biodegradation in the field has made very little progress in the last decade. As the general public and professionals perceived, knowledge of the possible hazards has resulted in stringent laws and reduced incentives for in situ bioremediation research, which has resulted in delayed development.

Various environmental factors that could control the degradation of the mixed contaminants were also involved. The elevation in antibiotic resistance has led to various health complications in humans and other living organisms. Apart from the detailed evaluation studies, several benchmark types of research have to be carried out to understand proper control methods. Antibiotic resistance and pollution are worldwide issues that are especially prevalent in underdeveloped countries, necessitating international cooperation, data exchange, and internationally uniform policy. The advancement in technology has led to the importance of bioinformatics, a new set of computational technologies combining information technology with biological sciences. It provides new hope in developing new and innovative technologies for solving the ever-increasing problem of mixed contaminants.

## Data Availability

Data are available from the authors on request (A.V.G.).
